# Case Report: Two Cases of Watershed Phenomenon in Mechanical Circulatory Support Devices: Computed Tomography Angiography Imaging and Literature Review

**DOI:** 10.3389/fcvm.2022.893355

**Published:** 2022-05-13

**Authors:** Guiying Du, Jiwang Zhang, Junbo Liu, Lijuan Fan

**Affiliations:** Department of Radiology, TEDA International Cardiovascular Hospital, Tianjin, China

**Keywords:** left ventricular assist devices, extracorporeal membrane oxygenators, case report, computed tomography angiography, watershed phenomeno, aortic root thrombus formation

## Abstract

Mechanical circulatory support (MCS) has become a processing technique used in end-stage heart failure (ESHF) because it can significantly improve survival and quality of life in patients with ESHF as either a transitional support therapy or a permanent replacement therapy before heart transplant. However, various potential complications associated with MCS need to be considered, especially aortic root thrombus formation. It’s critical to have an appropriate diagnosis of aortic root thrombus and “watershed” because the prognosis and treatment are different. Both “watershed” and aortic root thrombus formation can be characterized by computed tomography angiography. The CT manifestations of two patients who had MCS device implantation in our hospital (one with intra-aortic balloon pumps + extracorporeal membrane oxygenators, the other with left ventricular assist devices) were reported, and a literature review that recognized of “watershed” phenomenon in the aortic root was conducted.

## Introduction

There are an increasing number of therapies available for people with end-stage heart failure, particularly in the form of Mechanical circulatory support (MCS). It is intended to be used as a bridge to cardiac transplantation for patients with severe, refractory heart failure who have failed medical treatment (1). MCS devices such as intra-aortic balloon pumps (IABP), extracorporeal membrane oxygenators (ECMO), and left ventricular assist devices (LVAD) are commonly employed (1, 2).

For all this, various potential complications associated with MCS need to be taken into consideration, especially aortic root thrombus formation. Aortic root thrombosis is a rare complication that was recently recognized in continuous-flow left ventricular assist device therapy and is associated with significant mortality in MCS patients (3, 4). It can lead to serious consequences such as stroke, acute myocardial infarction, and even death (4). The “watershed,” also known as the “mixing zone” or “mixing cloud,” has been reported in ECMO implantation and is the specific location where the left ventricle cardiac output and ECMO flow mix. It is typically located along the ascending aorta (AAO), depending on the balance between the left ventricle cardiac output and the ECMO flow (5, 6). In clinical practice, an accurate diagnosis of aortic root thrombosis and “watershed” is essential because the prognosis and therapy are diverse. Both the “watershed” and the aortic root thrombus formation can be characterized by computed tomography angiography (CTA; 7–9). The CTA of two patients who had mechanical circulatory assist device implantation in our institution (one with IABP + ECMO, the other with LVAD) was evaluated. The two cases where the arc-shaped low-attenuation filling defect disappeared with the change of position represent a “watershed” phenomenon that can be seen in AAO.

## Case Presentation

### Case 1

#### Left Ventricular Assist Devices Implantation

A 56-year-old man with ischemic heart disease and a severely reduced ejection fraction underwent implantation of a “HeartCon” (TICH, Tianjin, China) continuous-flow left ventricular assist device. His medical history also included myocardial infarction, heart failure (NYHA IV), first-degree atrioventricular block, pulmonary arterial hypertension, and coronary artery bypass surgery. The LVAD has augmented the innate cardiac pumping function by creating an artificial blood flow conduit from the left ventricle to the descending aorta (DAO), thereby supplying the systemic circulation and maintaining proper tissue perfusion. The pump speed was 2,400 rpm. The blood pressure was 90/67 mmHg. The CTA was used for follow-up after 6 months of LVAD implantation.

### Case 2

#### Intra-Aortic Balloon Pumps and Extracorporeal Membrane Oxygenators Implantation

A 37-year-old man suffered from acute myocarditis, cardiogenic shock, arrhythmia-bradycardia, third-degree atrioventricular block, and paroxysmal tachycardia. His medical history also included acute liver injury, acute kidney injury, acute respiratory failure, and mixed acid-base balance disorder. The patient’s circulatory failure could not be repaired by IABP alone, and the veno-arterial ECMO (VA-ECMO) was implanted to stabilize circulatory collapse according to the indications for the use of cardiopulmonary support. The patient was admitted to the hospital for emergency coronary angiography, but the right coronary artery was not visualized. This examination was to assess the state of the right coronary artery.

## Computed Tomography Angiography Scanning

**FIGURE 1:**
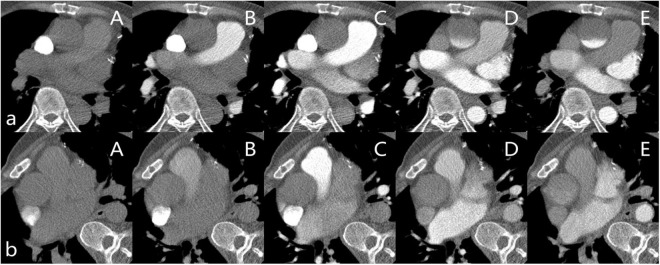
**(a)** Patient in the supine position. (A–E) are the slices of the AAO after the contrast agent injects 9s, 13s, 18s, 23s, and 28s in the Smart Prep Series. **(b)** Patient in the right lateral decubitus position. (A–E) are the slices of the AAO after the contrast agent injects 9s, 13s, 18s, 23s, and 27s in the Smart Prep Series.

The CTA of the two cases was performed using a prospective ECG-gated sequence in the arterial phase and iterative reconstruction with a 256-slice CT scanner (GE Revolution). Attenuation-based kV assist and automatic mA, as well as the bolus tracking technique, is used in CTA. A region of interest (1 cm^2^) is commonly positioned in the AAO for tracking the contrast bolus, and the acquisition is started with a 2-s delay after reaching a threshold of 300 HU. A non-ionic iodine contrast agent (Ioversol, Hengrui Jiangsu, China, iodine 350 mg/ml) was injected into an antecubital vein at 5.0 ml/s, followed by a 30 ml saline flush at the same rate. The patient was in the supine position at the first scan and changed to the right lateral decubitus position at the second scan (Figures 1a,b).

## Computed Tomography Angiography Imaging

### Case 1

**FIGURE 2:**
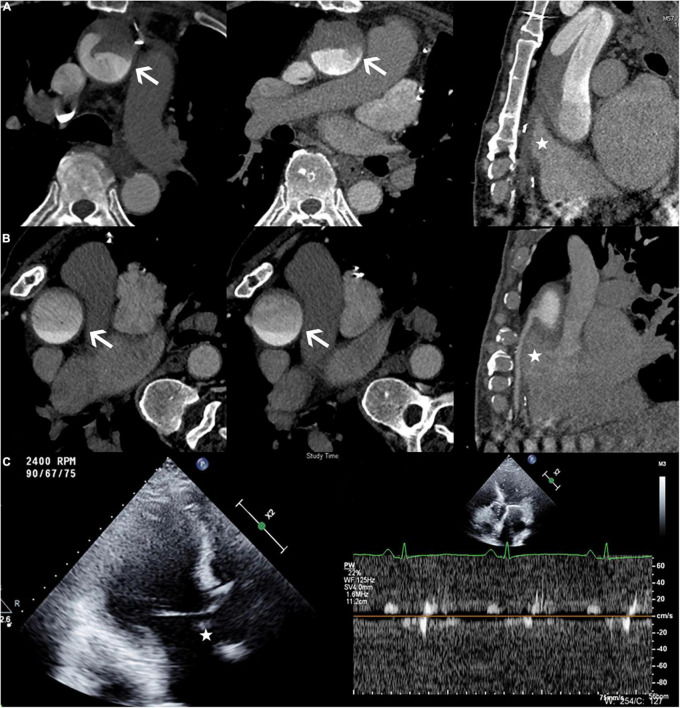
**(A)** Patient in the supine position. The axial view demonstrating an arc-shaped low-attenuation filling defect (white arrow) was seen in the root of the aorta, while the DAO was filling well. The multiplanar reformatted image from the CTA showed no contrast agent filling in the bypass of the right coronary artery (white star). **(B)** Patient in the right lateral decubitus position. The arc-shaped low-attenuation filling defect disappeared (white arrow), and the bypass of the right coronary artery filled well (white star). **(C)** The echocardiography (Philips EPIQ 7C; 45° reclining position; Three-chamber view) demonstrated the extremely low velocity of the aortic valve and no thrombus formation in the aortic root (white star).

In this case, an ECG-gated multidetector CTA showed a filling defect in the root of the aorta and no visualization of the bypass of the right coronary artery when the patient was in the supine position, while the DAO was filling well (Figure 2A). After turning the position to the right lateral decubitus position, the arc-shaped low-attenuation filling defect disappeared, and the bypass of the right coronary artery was filled with the contrast agent (Figure 2B). Transthoracic conventional echocardiography and two-dimensional spectral Doppler show that the blood flow velocity of the aortic valve was extremely low and there was no thrombus formation in the aortic root (Figure 2C).

### Case 2

**FIGURE 3:**
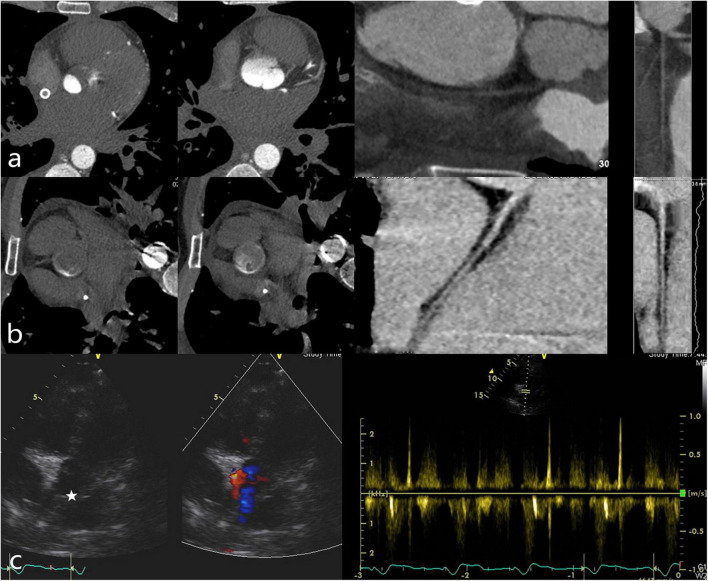
**(a)** Patient in the supine position. The axial view demonstrated an arc-shaped low-attenuation filling defect (“watershed” phenomenon) in the root of the aorta while the DAO was filling well. The multiplanar reformatted image from the CTA showed no contrast agent filling in the right coronary. **(b)** Patient in the right lateral decubitus position. The arc-shaped low-attenuation filling defect disappeared and the right coronary filled well. (Left dominant coronary artery; **(c)** The echocardiography (GE Vivid E95; 45° reclining position; Five-chamber view) demonstrated the extremely low velocity of the aortic valve and there was no thrombus formation in the aortic root (white star).

In this case, the ECG-gated multidetector CT angiography showed a filling defect in the root of the aorta with no visualization of the right coronary artery when the patient was in the supine position, while the DAO was filling well (Figure 3a). After turning the patient to the right lateral decubitus position, the arc-shaped low-attenuation filling defect disappeared. In addition, changing the patient’s scanning position resulted in the good filling of the right coronary artery that was not filled on scanning in the supine position (Figure 3b). Transthoracic conventional echocardiography and two-dimensional spectral Doppler show that the blood flow velocity of the aortic valve was extremely low and there was no thrombus formation in the aortic root (Figure 3c).

## Follow-Up and Outcomes

While awaiting a heart transplant, the vital signs of Case 1 have stabilized and his circulatory function has improved.

The patient from Case 2 has healed. The left ventricular ejection fraction has gone back to normal, and the blood circulation has stabilized.

## Discussion

In two of our patients with MCS implantation, there was a low-attenuation filling defect in the aortic root on CTA, which disappeared after altering the patient’s position, thus confirming that it was not a true thrombosis but a “watershed” in the aortic root. This “watershed” of the aortic root is caused by the opposite bleeding between MCS flows and the left ventricle. Meanwhile, several other complications of MCS, such as thrombus formation, also deserve our attention. Because the prognosis and therapy for aortic root thrombosis and “watershed” vary, it’s vital to get an accurate diagnosis.

## “Watershed” Phenomenon of Mcs on Computed Tomography Angiography

Extracorporeal membrane oxygenators is commonly utilized as a bridge to recovery and the installation of ventricular assist devices in refractory cardiogenic shock, and the veno-arterial configuration is used for cardiovascular support (10–12). In most ECMO patients, the left ventricle maintains some residual output and so provides an antegrade blood flow to the systemic circulation *via* the aortic valve (6). But the flow rate at the aortic root is extremely low (13). In addition, ECMO flow is in contrast to antegrade native cardiac output. Blood stasis can develop as a result of retrograde blood flow in the AAO during VA-ECMO, which can lead to intracardiac or extracardiac thrombosis (14). The LVAD is able to unload the left ventricle effectively and provide the heart with real rest, and the aortic valve is almost in a closed state (15). Due to the location of the LVAD outflow cannula, the local blood flow pattern might be influenced, resulting in a stagnant flow zone in the backflow portion. Such stagnation zones may cause the formation of thrombosis (16).

Computed tomography angiography is a widely available and capable technique that can be employed to evaluate thrombosis in the aorta in patients with MCS (7, 17, 18). In our center, two patients who got IABP + ECMO and LVAD surgery also underwent CTA. The aortic root contrast medium fills slowly on CTA in which the blood displays a filling defect in the root of the aorta with no visualization of the right coronary artery or coronary artery bypass while the patients were supine. The arc-shaped low-attenuation filling defect disappeared when the position was changed to the right lateral decubitus position and the right coronary artery or the bypass of the coronary artery was filled with the contrast agent, both of which displayed the “watershed” phenomenon on CTA in the root of the aorta. The changes strongly demonstrate that the arc-shaped low-attenuation filling defect in the root of the aorta is not a thrombus formation. Meanwhile, echocardiography reveals that no thrombus formation occurs even if the pressures and flows of aortic roots are extremely low.

The pressures and flows of the left ventricle outflow trace are low, and the oxygen content of the blood coming from the left ventricle is unknown. The “watershed” phenomenon may put the coronary artery and supra-aortic branches at risk of poor perfusion and hypoxemia (5, 8). Thus, cardiac ischemia may occur in patients receiving MCS support. In our cases, there was no contrast agent filling in the right coronary (the bypass of the right coronary artery) on CTA, which demonstrated that perfusion and flow of the right coronary (the bypass of the right coronary artery) were inadequate. With insufficient blood supply, the myocardium could be injured. Right heart failure is a complication of MCS, particularly in LVAD patients, and the insufficient blood supply to the coronary could be one of the causes.

Furthermore, if MCS flow perfusion levels do not reach the origins of supra-aortic arteries, brain circulation may be limited. The first case shows that a portion of the supra-aortic branches was receiving blood from the LVAD circuit, implying that brain circulation may be restricted. The supra-aortic branches are not reached by the scanning scope in case two.

## Aortic Root Thrombus Formation

The MCS implantation such as ECMO and LVAD has various severe complications, including thrombosis. The majority of thrombosis instances documented are ventricular, while intra-aortic thrombosis is extremely rare, with only a few examples reported in the literature, and the mortality of patients is extremely high (14, 15, 19). Devastating complications such as strokes, acute myocardial infarction, or even death can be caused by thrombus deposition in the aortic root (4). Not only is there decreased blood flow velocity in the aortic root, but the increased levels of clotting factors also lead to an increased risk of thrombosis in MCS patients (12, 20–22). The thrombus in the aortic root shows a permanent filling defect that does not alter with the filling and clearance of the contrast agent (15). The “watershed” phenomenon has no additional effect on the coagulation status or the stability of blood circulation.

To our knowledge, the “watershed” phenomenon has never been mentioned on LVAD. What’s more, this is the first report of the discriminate “watershed” phenomenon and thrombus formation with MCS implantation verified by CTA. The diagnosis of the aortic root “watershed” phenomenon described here may lead to a false diagnosis. When a patient undergoes a CTA scanning, altering the right lateral position for supplementary scanning is critical for detecting this sign and distinguishing it from thrombosis.

## Conclusion

The signs of the “watershed” phenomenon are not common in patients with MCS, and it is necessary for radiologic technologists and radiologists to accumulate experience in scanning and diagnosis, as well as pay attention to distinguishing it from thrombosis. Accurate identification of aortic root thrombosis is critical for both clinical and postoperative management. With proper evaluation and diagnosis, patients can have favorable prognoses.

## Data Availability Statement

The original contributions presented in the study are included in the article/supplementary material; further inquiries can be directed to the corresponding author/s.

## Ethics Statement

All subjects gave written informed consent in accordance with the Declaration of Helsinki. The protocol was approved by the institutional review board (IRB) of TEDA International Cardiovascular Hospital. The present study involved no potential risk to patients.

## Author Contributions

GD and LF: responsibility for the integrity of the work as a whole, from inception to finished article. GD and JL: acquisition of imaging. GD and JZ: analysis and interpretation of imaging. All authors were involved in drafting the article or revising it critically for important intellectual content, and all authors approved the final version to be published.

## Conflict of Interest

The authors declare that the research was conducted in the absence of any commercial or financial relationships that could be construed as a potential conflict of interest.

## Publisher’s Note

All claims expressed in this article are solely those of the authors and do not necessarily represent those of their affiliated organizations, or those of the publisher, the editors and the reviewers. Any product that may be evaluated in this article, or claim that may be made by its manufacturer, is not guaranteed or endorsed by the publisher.
